# Supplementary material for: A deep learning-based toolkit for 3D nuclei segmentation and quantitative analysis in cellular and tissue context
Source: Development. 2024 Jul 18;151(14):dev202800. doi: 10.1242/dev.202800 (PMC11273294; doi:10.1242/dev.202800)
Supplement: Supplementary information [file develop-151-202800-s1.pdf]

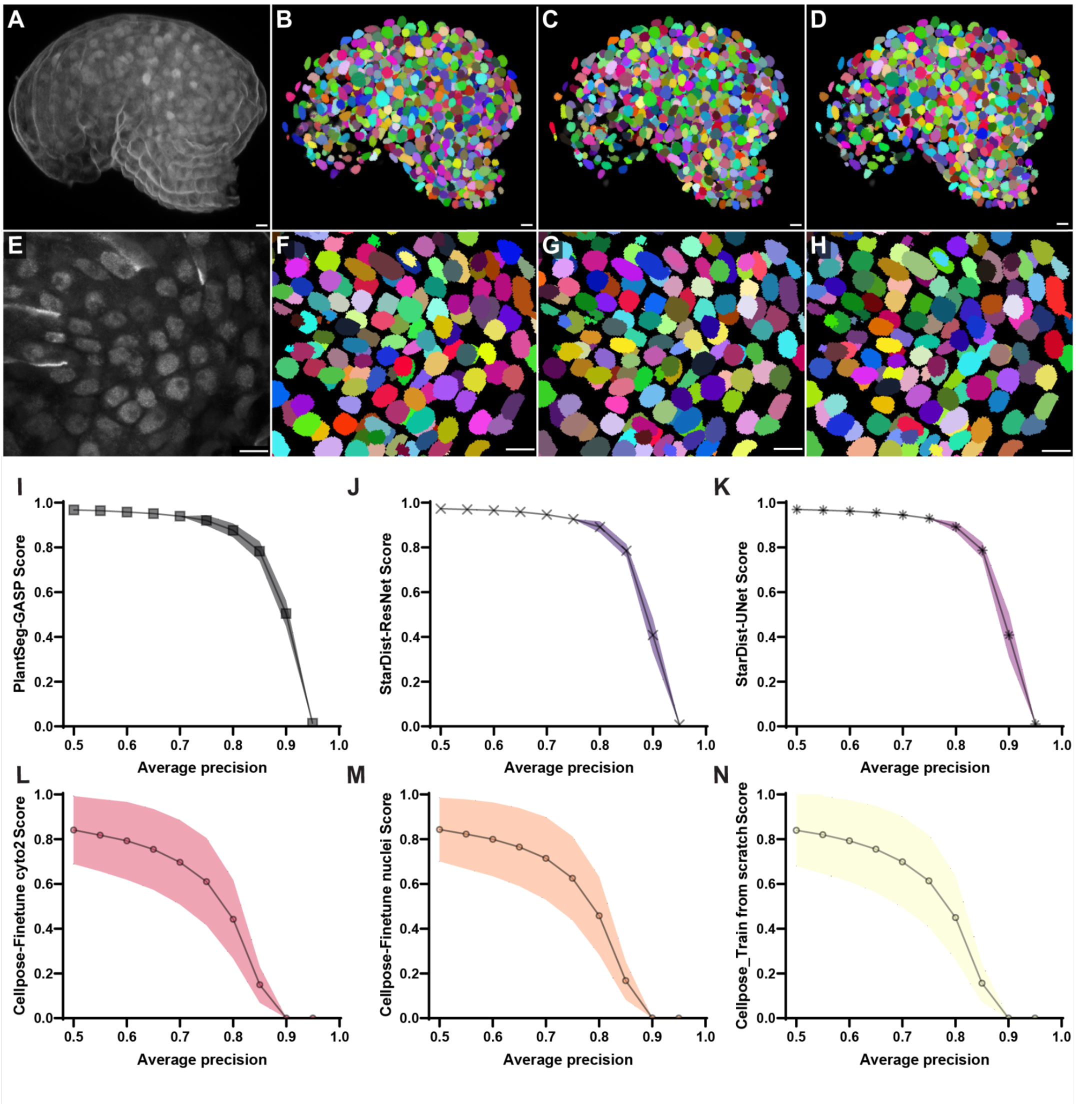

Fig. S1. Qualitative analysis of Cellpose models and average precision scores for all trained models. Qualitative comparison displaying the Arabidopsis ovule testing dataset 1135 (N1 dataset) with trained model (Model-5) using four other training datasets. (A, E) Raw weak TO-PRO 3 iodide nuclei stain in 3D and 2D view respectively. (B, F) Results of Cellpose fine tuned cyto 2 model in 3D and 2D view respectively. (C, G) Results of Cellpose finetune nuclei model in 3D and 2D view respectively. (D, H) Results of Cellpose model trained from scratch model in 3D and 2D view respectively. (I-N) Quantitative comparison of segmentation results using different proposed training methods. Graphs represent the mean and standard deviation values using five fold trained models approach. (I) Results of PlantSeg GASP nuclei segmentation. (J) Results of StarDist ResNet model training. (K) Results of StarDist Unet model training. (L) Results of Cellpose fine tuned cyto 2 model training. (M) Results of Cellpose fine tuned nuclei model training. (L) Results of Cellpose model trained from scratch. Scale bars: 5µm.

Development • Supplementary information

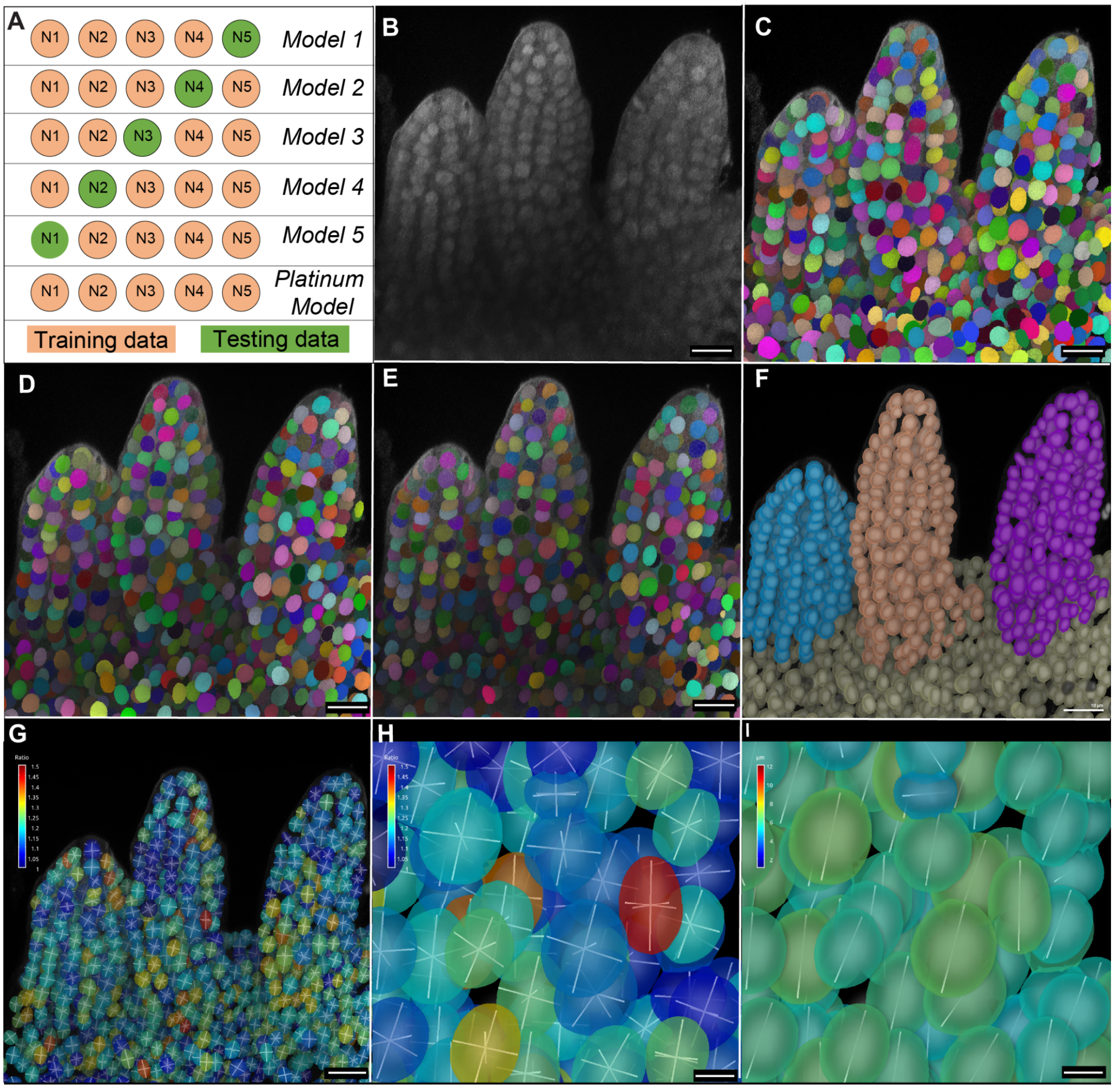

Fig. S2. Outline of model training strategy and qualitative comparison of 3D nuclear segmentation results using the StarDist-ResNet and PlantSeg platinum models. Young Arabidopsis ovule dataset 1221, unused for all training, was used as a testing dataset. (A) Outline of the 5-fold training approach used for scoring different listed methods and training the platinum model. N1- N5 represent raw image datasets used for training and testing purposes. N1 (dataset 1135), N2 (dataset 1136), N3 (dataset 1137), N4 (dataset 1139), N5 (dataset 1170). Model 1 was trained on N1-N4 dataset and tested with N5 dataset. Model 2 was trained on N1, N2, N3, N5 and was tested on N4. Models 3, 4 and 5 were generated in a similarly interactive manner. The platinum model (final model) was trained on all datasets. Green and light-brown colors indicate training and testing models, respectively. (B) Raw weak TO-PRO 3 iodide nuclei stain in 3D view. (C) Ground truth nuclei segmentation. (D) Results of StarDist-ResNet platinum model trained on all five datasets. (E) Results of PlantSeg platinum model trained on all five datasets. (F) 3D rendering in MorphographX with 3D meshes representing the nuclei segmentation from StarDist ResNet model. Different colors indicate the annotations of different young ovule primordia and the placenta tissue performed on the 3D nuclei mesh. (G, H) Elongation of nuclei is

Development • Supplementary information

quantified in MGX and values are indicated with a heatmap ranging from 1-1.5 (ratio of max by mid axis length). (H) A zoomed-in region displaying differently elongated nuclei. White lines inside nuclei indicate the max-mid-min axes from the nuclei centroid. (I) Length of nuclei is quantified along the max axis as a heatmap. The white line indicates the max axis orientation. Heatmap range 0-12µm. Scale bars: (B-G) 10µm; (H,I) 2µm.

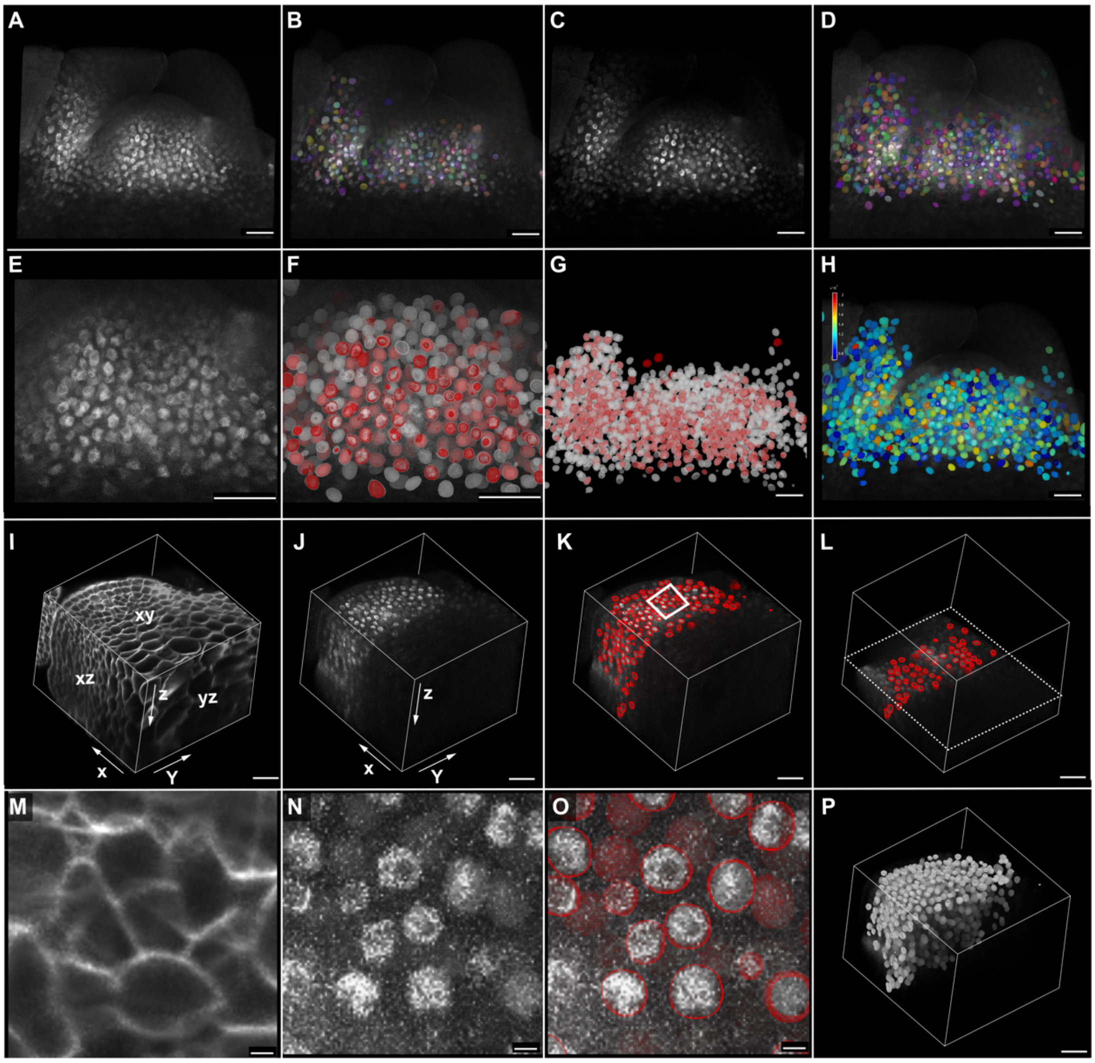

Fig. S3. Applicability of the StarDist-ResNet_3Dnuc_platinum model for segmentation of a more taxing dataset after raw image preprocessing. (A) Raw confocal image of an Arabidopsis shoot apical meristem (SAM) cleared and imaged using a 40x 1.3NA Gly objective. Raw image depicts the expression of the pFD:3xHA-mCHERRY-FD reporter. Image from (Cerise et. al. 2023). (B) Nuclei mesh view of raw image segmented using the StarDist-ResNet-platinum model. (C) Raw data preprocessed by performing a 3D median blur (1x 1 x 1 μm^3 in xyz directions). (D) Nuclei mesh view of processed image segmented using the StarDist-ResNet-platinum model. (E, F) A zoomed 3D clip plane view. (E) Raw data. (F, G)

Development • Supplementary information

Comparison of segmentation using the raw data and the preprocessed data. Red color represents the nuclei segmented with both raw and preprocessed images. White color represents the nuclei undetected by just the raw data segmentation, those were segmented using the preprocessed data. (H) Nuclear signal quantification from these faint raw data after performing the preprocessing and nuclei segmentation using the StarDist-ResNet-platinum model. Signal quantification was performed in MorphographX. Colors scale from blue to red indicate the minimum and maximum signal intensity of the reporter line. (I-L, P) A 3D cropped region of the stack visualizing xy, xz and yx planes to qualitatively visualize the nuclear segmentation in 3D. The xy plane represents a frontal section of a SAM. (I) Raw SR2200 signal showing the image to be of high quality for this channel. (J) View of raw signal used for segmentation in 3D. (K) Red outlines represent segmented nuclei from the signal after preprocessing. (L) Image revealing quality of segmentation from a deeper tissue layer. (M-O) A zoomed-in region represented by a bounding box in (K) showing the challenging and noisy nuclei signalchalle egmentation. (P) 3D view3D vill segmented nuclei in this image. Scale bars: (A-L, P) 20 µm; (M-O) 2 µm.

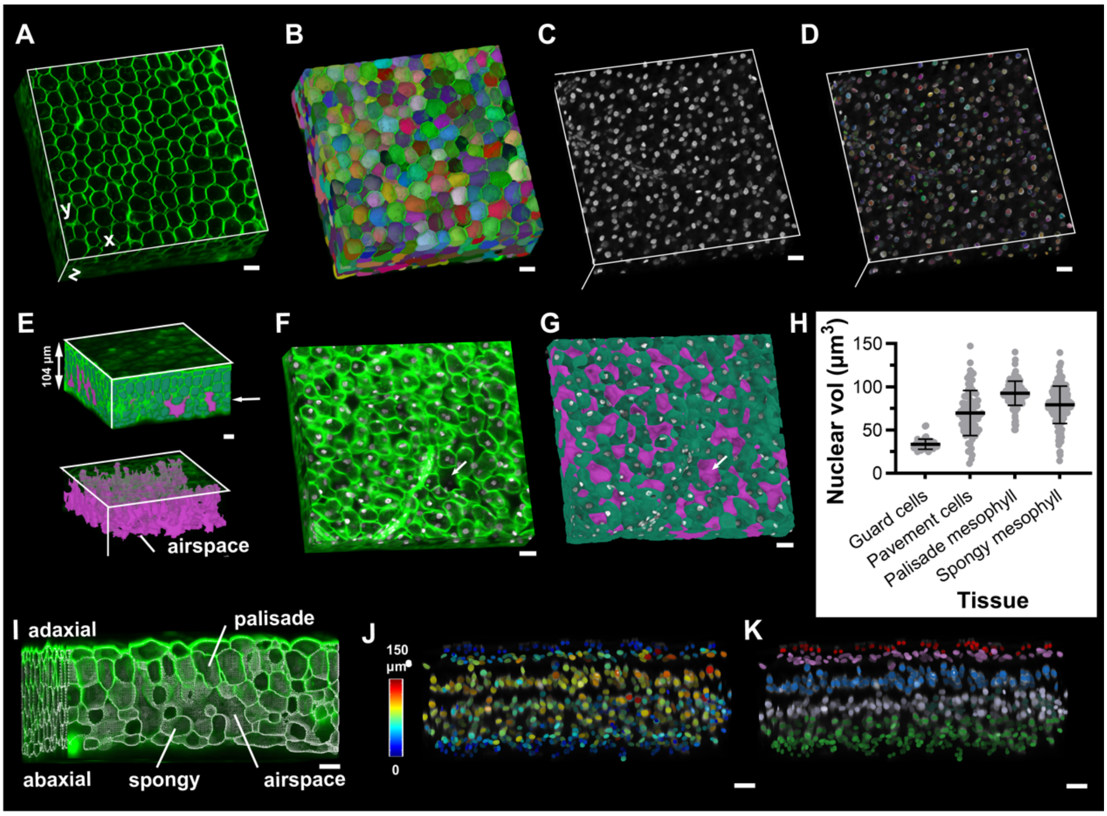

Fig. S4. Cellular and nuclear analysis of a 100 µm thick 3D volume of a region of leaf 2 of a 14-day-old Arabidopsis plant. (A) Raw SR2200 cell wall stain, (B) Cell segmentation overlaid with raw cell wall stain. (C) Raw H2B::TdTomato nuclei, (D) 3D Nuclear segmentation úsing StarDist-ResNet model, (E) 3D volume of the sample. Cells and air spaces are annotated in the cell mesh obtained from segmented stack. The arrow indicates the z-slice of the spongy mesophyll shown in (F,G). Bottom panel shows the 3D airspace volume extracted from the cell mesh. (F,G) Overlay of an XY sectioned 3D view showing spongy mesophyll cell morphology and cell and airspace segmentation. (F) Raw cell wall and H2B nuclei image are overlaid. (G) Corresponding segmented mesh view after annotation of cells and airspaces. The nuclei image is overlaid. Arrows indicate airspace without nuclei. (H) Graph depicting the nuclear volumes sampled from different leaf tissues. (I) 3D transverse view showing the overlay of cell wall image in green, and the outlines of the cells extracted from the cell segmentation mesh in white. The XZ axis of the sample is shown with the annotation of different tissues and airspaces. (J) Heatmap of nuclear volumes. (K) Cell-type/tissue-type based classification of nuclei distinguished by color. The green nuclei at the bottom belong to abaxial epidermis, some spongy mesophyll cells, and abaxial guard cells. Due to the difficulty of separating these nuclei into different tissues, they are not shown in the graph (H). Scale bars: 20 µm.

Development • Supplementary information

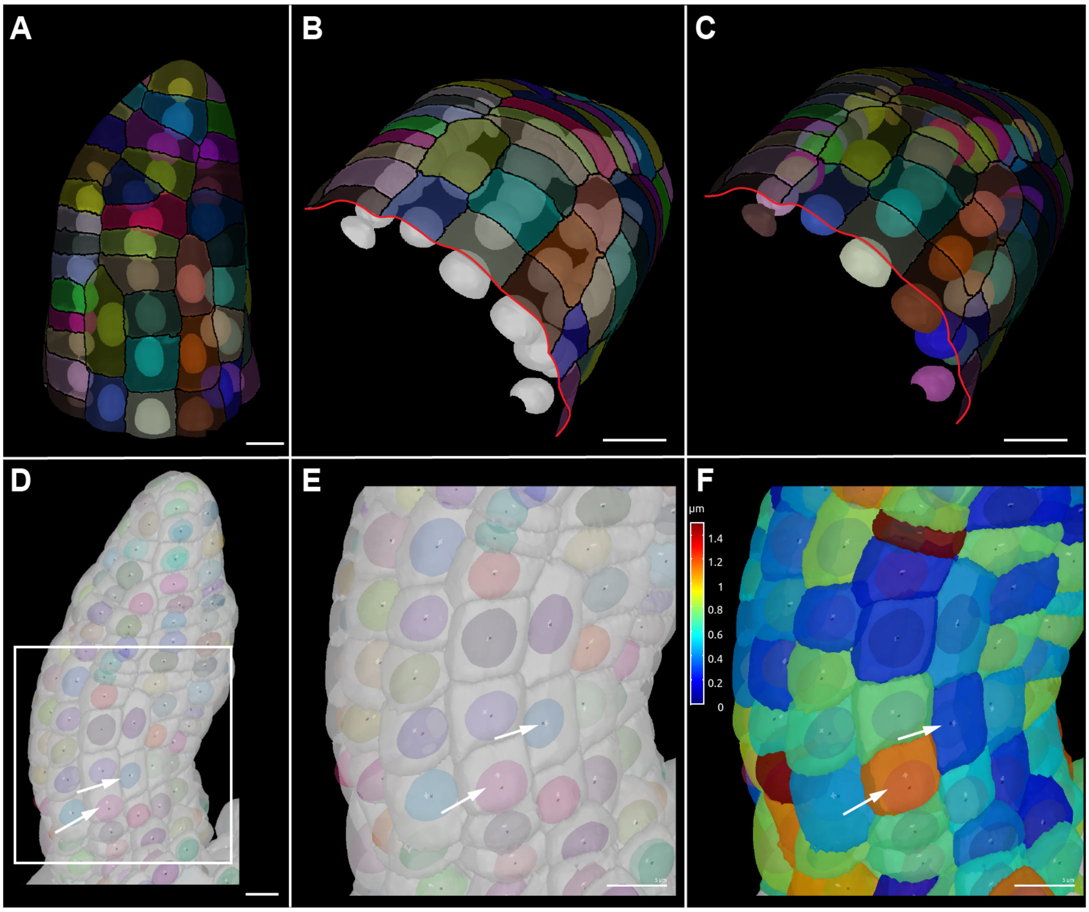

Fig. S5. Linking 2.5D cells to 3D nuclei and distance quantification between cell and nuclei centroid. (A-C) Linking 2.5D cells to 3D nuclei. (A) Frontal view of the ovule primordium. 3D nuclei with linked cell labels from corresponding 2.5D cells above. (B) Side view of a zoomed region with unlinked 3D nuclei in white and segmented 2.5D cells with colors. (C) Same view from (B) with 3D nuclei linked to 2.5D cells as indicated by the same color. Red outline indicates a section through the 2.5D surface mesh. (D-F) Euclidean distance quantification between 3D cells and 3D nuclear centroids. Black and white dots represent 3D nuclei and 3D cell centroids, respectively. Transparent overlay of 3D cell mesh and 3D nuclei mesh. (D-E) Cell mesh in white color, nuclei with the segmentation label for visualization of centroids. (E) Zoomed region from D indicating difference in the position of nuclei within cells. (F) Same view from (E) with heatmap quantification of Euclidean distance between cell and nuclei centroid. Heatmap scale ranges from 0-1.5μm. Arrows indicate representative cells with high and low distance between cell and nuclei centroids. Scale bars: 5μm.

Development • Supplementary information

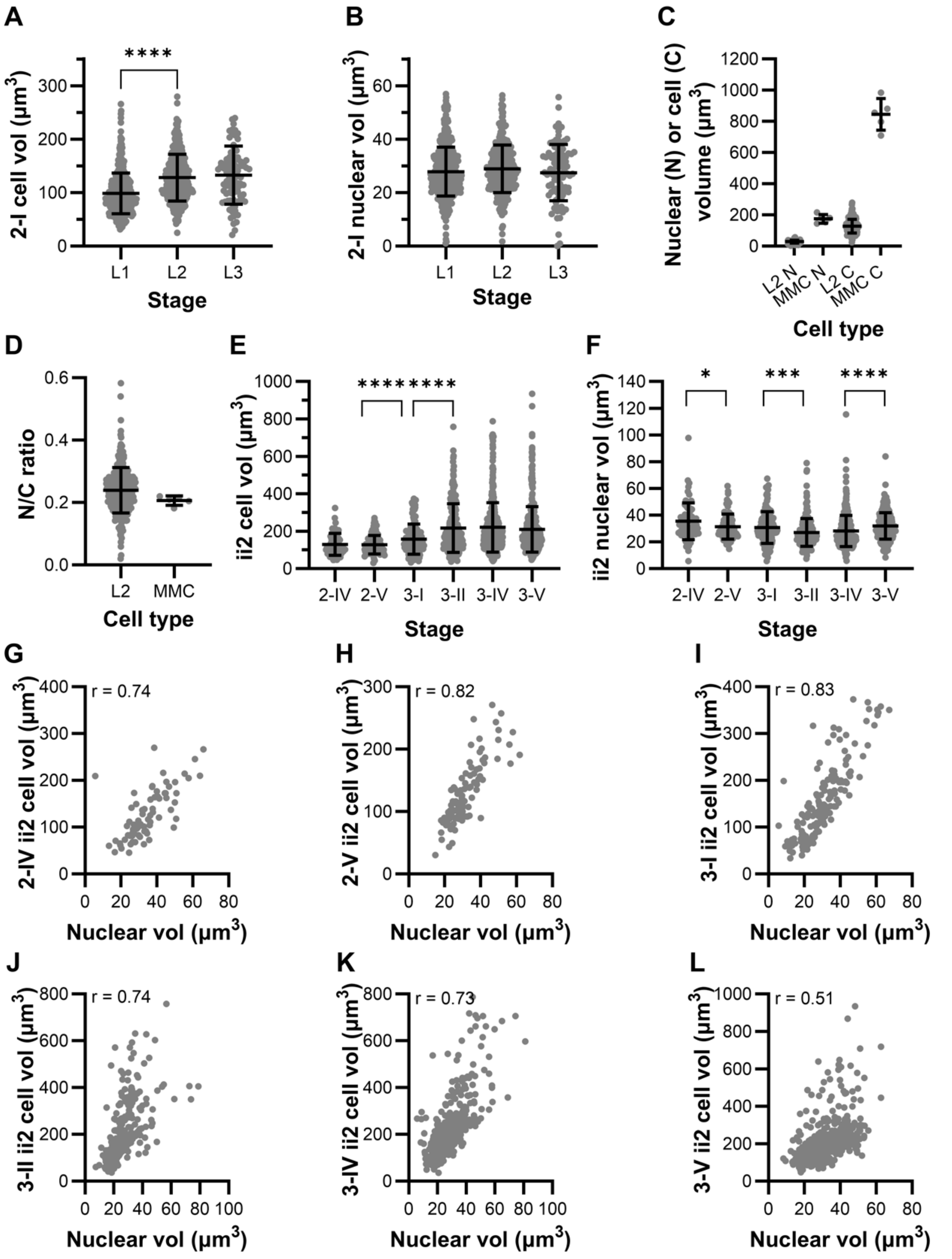

Fig. S6. Nuclear volume and cell volume linking and correlation analysis. (A) Plot showing stage 2-I radial layer-specific cell volumes. (B) Plot showing stage 2-I radial layer-specific nuclear volumes. (C) Plot depicting the nuclear and cell volumes of the L2 cells (without the MMCs) and the MMCs. (D) Plot showing the N/C ratios of the L2 cells (without the MMCs) and the MMCs. (E) Plot showing stagewise cell volumes for the outer layer of inner integument (ii2). (F) Plot showing stagewise nuclear volumes for the outer layer of inner integument (ii2). (G-L) Plots showing correlation

Development • Supplementary information

between nuclear and cell volumes of ii2 at different stages of ovule development from 2-IV up to 3-V along with the respective Pearson correlation coefficients, r: (G) 2-IV, (H) 2-V, (I) 3-I, (J) 3-II, (K) 3-IV, and (L) 3-V. Asterisks represent statistical significance (ns, $p \geq 0.5$; *, $p<0.05$; **, $p<0.01$, ***, $p<0.001$; ****, $p<0.0001$; Student's t-test).

Table S1. Comparative analysis of different model performance when input image is resampled to isotropic compared to original dimension.

**Tool**	**Model**	**resampling**	**mAP ± STD**
PlantSeg	GASP	original	76.82% ± 1.98%
PlantSeg	GASP	isotropic	66.41% ± 1.85%
PlantSeg	GASP	isotropic 3D rot	66.39% ± 2.03%
Cellpose	nuclei	original	31.94% ± 12.18%
Cellpose	nuclei	isotropic	39.46% ± 12.51%
StarDist	ResNet	original	76.6% ± 1.73%
StarDist	ResNet	isotropic	64.86% ± 0.93%
StarDist	ResNet	isotropic 3D rot	61.17% ± 1.61%

Segmentation of the test dataset in original and isotropic resampled dimensions were performed separately using each of the listed models and mean average precision for different methods are scored compared to their respective human proofread ground truth. For model training we recommend to use the 3D data in its original form and only preprocess if necessary, e.g. rescale to fit field of view for StarDist and use isotropic slices for Cellpose.

Development • Supplementary information

Table S2. Comparison of Cellpose original nuclei model, TWANG and PlantSeg cellpose hybrid method with Cellpose original nuclei and the fine tuned model

**Method**	**Instance segmentation**	**mAP score**
Cellpose original nuclei	Cellpose original nuclei	9.76%
TWANG	TWANG	14.41%
PS-CP	Cellpose original nuclei	57.82%
PS-CP	Cellpose nuclei fine tuned	25.65%

Mean average precision is scored for different methods compared to their respective human proofread groundtruth. Dataset N1 (1135) is used for quantifying the scores.

Table S3. Detailed quantification of AP scores for evaluation of segmentation

Available for download at
https://journals.biologists.com/dev/article-lookup/doi/10.1242/dev.202800#supplementary-data
